# First Draft Genome Sequence of a Clinical Strain of *Nocardia cerradoensis*

**DOI:** 10.1128/genomeA.00551-17

**Published:** 2017-09-28

**Authors:** Gema Carrasco, Sara Monzón, Pilar Jiménez, Isabel Cuesta, Joaquín Bartolomé-Alvarez, Sylvia Valdezate

**Affiliations:** a Reference and Research Laboratory for Taxonomy, Department of Bacteriology, National Centre of Microbiology, Instituto de Salud Carlos III, Majadahonda, Madrid, Spain; b Laboratory of Bioinformatics, Common Scientific Technical Units, Instituto de Salud Carlos III, Majadahonda, Madrid, Spain; c Genomics Laboratory, S. G. for Applied Services, Training and Research, Instituto de Salud Carlos III, Majadahonda, Madrid, Spain; d Microbiology Department, University Hospital of Albacete, Albacete, Spain

## Abstract

This paper reports the first draft genome sequence for a strain of *Nocardia cerradoensis* obtained from an immunocompetent patient with a knee infection. The 8.2-Mb genome has 8,329 coding sequences, including intrinsic resistance genes, biosynthetic gene clusters for polyketide synthase and nonribosomal peptide synthase, virulence genes, and prophages.

## GENOME ANNOUNCEMENT

*Nocardia* species are environmental bacteria that cause severe opportunistic infections of the lung, skin, and central nervous system, although mainly in immunocompromised patients. Of 110 recognized species of this genus (http://www.bacterio.net/), more than half are described as human pathogens. *Nocardia cerradoensis* was initially described in Brazilian soil (1); as a pathogen it was first noticed causing disseminated infection in a patient who had received a kidney graft (2). Although *N. cerradoensis* has been proposed as an emerging global pathogen (3), no genome of an *N. cerradoensis* clinical strain has previously been sequenced. Here, we describe the draft genome sequence of *N. cerradoensis* CNM20130759, isolated from a postinfiltration cutaneous infection on the knee of a 52-year-old immunocompetent woman (4). The antimicrobial profile of this isolate fitted that describ*e*d for the *Nocardia nova* complex pattern (5). The 16S rRNA and *gyrB* genes, and the multilocus sequence type (6), were most similar to those of *N. cerradoensis* W9747^T^ (99.9%, 99.5%, and 99.6% similarities, respectively).

A paired-end library (Nextera-XT DNA library preparation kit, Illumina 1.9) was adapted and sequenced at 2 × 150 in the Illumina NextSeq 500 platform. After quality control analysis using fastQC v.0.11.3 (http://www.bioinformatics.babraham.ac.uk/projects/fastqc/) and Trimmomatic v.0.36 software (7), ∼4.9 million paired-end reads of 50 to 151 bp were retrieved. Assembly of the reads was performed using SPAdes v.3.8.0 (8), and the quality of the assembly was evaluated using QUAST software (9).

The genome was found to be 8,193,765-bp long, with a G+C content of 68.16% (150 contigs of ≥500 bp; maximum, 1,035,523 bp; *N*_50_ value, 394,043 bp). The assembled sequences were annotated using Prokka 1.12-beta software (10), which predicted 8,406 genes (8,329 protein-coding sequences, 5 rRNA genes, 1 transfer-messenger RNA [tmRNA] gene, and 71 tRNA genes) and 543 signal peptides. Among the protein-coding genes, 2,060 were unique and 704 repeated 2 to 41 times. Multiple antibiotic resistance and efflux pump genes [*acc*(6′)-I, *blaI*, *drrA*,* drrB*,* drrC*,* emrY*,* inhA*,* mdtG*,* mdtK*,* mdtH*,* mdtL*,* mmr*, NCRCNM_01156, NCRCNM_01157, NCRCNM_01690, NCRCNM_01779, NCRCNM_06233, NCRCNM_06526, *norM*,* stp*, *tetR*, and *yfmO*, among others], antiseptic genes (*sugE* and *qacA*), metalloproteinase genes (*tldD*, *yflN*, *ycnJ*, and *merR*), and integrase genes (Int-Tn 1 to 3) were detected, but no clustered regularly interspaced short palindromic repeats (CRISPR) were found. One intact prophage (with *att*L/*att*R sites) and five incomplete prophages were identified by PHAST software (11), representing 0.84% of the genome. Neither SRST2 (12) nor ARGannot (13) software detected any plasmid or acquired resistance genes.

Virulence genes for *mce*2 (mammalian cell invasion), hemolysin, superoxide dismutase, catalase-peroxidase, cutinase, esterases, hemagglutinin, the antigen 85 complex family, ESX-1, and sortase A (among others) were all detected; some of these have been previously described for *Nocardia farcinica* (14). Biosynthetic gene clusters such as 12 nonribosomal peptide synthases (NRPS), 8 polyketide synthases (PKS-I-III), 4 terpene cyclases, 6 NRPS, and 2 unique PKS-I genes were found using antiSMASH software (15).

This clinical strain showed 99.4% similarity, by ANI software (16), to the *N. cerradoensis* W9747^T^ draft genome (a soil bacterium), which has a length of 7.6 Mbp, a G+C content of 68.16%, 7,216 protein-coding genes (8 rRNAs), and 1 CRISPR. Increased understanding of the differences between the human and environmental strains expands our knowledge of *N. cerradoensis*.

### Accession number(s).

This whole-genome shotgun project has been deposited at DDBJ/ENA/GenBank under the accession number NGAF00000000. The version described in this paper is version NGAF01000000.
